# Facilitating or disturbing? An investigation about the effects of auditory frequencies on prefrontal cortex activation and postural sway

**DOI:** 10.3389/fnins.2023.1197733

**Published:** 2023-06-22

**Authors:** Valeria Belluscio, Giulia Cartocci, Tommaso Terbojevich, Paolo Di Feo, Bianca Maria Serena Inguscio, Marco Ferrari, Valentina Quaresima, Giuseppe Vannozzi

**Affiliations:** ^1^Department of Movement, Human and Health Sciences, Interuniversity Centre of Bioengineering of the Human Neuromusculoskeletal System, University of Rome “Foro Italico”, Rome, Italy; ^2^Fondazione Santa Lucia, Rome, Italy; ^3^Department of Molecular Medicine, Sapienza University of Rome, Rome, Italy; ^4^BrainSigns Ltd, Rome, Italy; ^5^Department of Human Neuroscience, Sapienza University of Rome, Rome, Italy; ^6^Department of Life, Health and Environmental Sciences, University of L’Aquila, L’Aquila, Italy

**Keywords:** biomechanics, functional near-infrared spectroscopy, balance, single-leg stance, double-leg stance, inertial sensors, wearable technology, auditory cue

## Abstract

Auditory stimulation activates brain areas associated with higher cognitive processes, like the prefrontal cortex (PFC), and plays a role in postural control regulation. However, the effects of specific frequency stimuli on upright posture maintenance and PFC activation patterns remain unknown. Therefore, the study aims at filling this gap. Twenty healthy adults performed static double- and single-leg stance tasks of 60s each under four auditory conditions: 500, 1000, 1500, and 2000 Hz, binaurally delivered through headphones, and in quiet condition. Functional near-infrared spectroscopy was used to measure PFC activation through changes in oxygenated hemoglobin concentration, while an inertial sensor (sealed at the L5 vertebra level) quantified postural sway parameters. Perceived discomfort and pleasantness were rated through a 0–100 visual analogue scale (VAS). Results showed that in both motor tasks, different PFC activation patterns were displayed at the different auditory frequencies and the postural performance worsened with auditory stimuli, compared to quiet conditions. VAS results showed that higher frequencies were considered more discomfortable than lower ones. Present data prove that specific sound frequencies play a significant role in cognitive resources recruitment and in the regulation of postural control. Furthermore, it supports the importance of exploring the relationship among tones, cortical activity, and posture, also considering possible applications with neurological populations and people with hearing dysfunctions.

## 1. Introduction

Postural control, the act of maintaining, achieving, or restoring a state of balance during any posture or activity (Pollock et al., 2000), is a complex motor skill derived from the interaction of visual, somatosensory, and vestibular systems (Nashner, 1973; Horak, 2006; Maurer et al., 2006), with the latter usually considered as major input (Zhong and Yost, 2013). Scientific evidence also suggests that auditory information plays a role in postural control regulation in healthy people and in those with pathological conditions (Zhong and Yost, 2013; for a recent review see Schaffert et al., 2019), but it is still unclear to what extent (Ross et al., 2016). Phonoreceptors and the vestibular organ are anatomically and functionally mutually connected: the auditory organ receives sound stimuli in the form of a wave of air density, which can possibly influence the postural regulation (Siedlecka et al., 2015). In the presence of hearing or vestibular dysfunctions, the auditory system provides balance-related cues which allow body sway to decrease by almost 41% (Stevens et al., 2017). However, in studies involving healthy control participants, findings are contrasting, with increased, reduced, or no change in body sway measured while exposed to different auditory stimuli (Juntunen et al., 1987; Raper and Soames, 1991; Easton et al., 1998; Tanaka et al., 2001; Russolo, 2002; Park et al., 2011; Yu et al., 2015, 2016; Gandemer et al., 2017; Thomas et al., 2018; Lubetzky et al., 2020). Discrepancy in results could be explained by the different sound sources (i.e., administration through headphones, background noise, localization) (Gandemer et al., 2016) and their characteristics (i.e., loudness, frequency) (Fletcher and Munson, 1933; Petersen et al., 1996). Therefore, to better reveal the relationship between auditory stimuli and postural control, there is a need to explore the role of an auditory stimulus based on its main characteristics. Different authors tried to get deeper into the sound-postural control associations by focusing on sound frequency (or tone), defined as the subjective feeling of the sound mainly related to the frequency of acoustic waves (the higher the acoustic wave’s frequency, the higher the tone) (Yu et al., 2016). Some authors observed that low-frequency sounds can reduce postural sway compared to no sound condition (Easton et al., 1998; Park et al., 2011), with the length of the sway path increasing as the frequency of sound increases. On the other hand, several results seem to point out that higher frequencies (> 1000 Hz) improve motor performance (Raper and Soames, 1991; Russolo, 2002; Yu et al., 2016). In Russolo (2002), the author observed increased postural oscillations associated with acoustic stimulations at 250 and 500 Hz in almost all the study participants, suggesting that low frequencies elicited higher oscillations. In Raper and Soames (1991), static 250 Hz pure tone and general background conversation at 65 decibels (dB) produced a destabilizing effect on balance control. Differently Yu et al. (2016), exploring the effects of 500, 1000, 1500, and 2000 Hz frequencies on healthy people’s lower limb motor function during different motor tasks, reported that the highest frequencies improve performance. Thus, results are controversial, and the situation is even more complicated if we consider that not only type of sound and frequency are associated with postural sway, but also the pleasantness/unpleasantness of an auditory stimulus. For example, Chen and Qu (2017) found that unpleasant auditory stimuli were associated with larger postural sways and that the average ratings of perceived disturbance significantly increased as the frequency and the sound pressure level (SPL) increased. Another aspect that should be considered for explaining the association between sound characteristics and postural control regulation pertains to the role of attention in sensory integration. Despite little conscious effort is required to maintain the upright stance, several brain structures play a crucial role in balance maintenance (Takakusaki, 2017; St George et al., 2021). The cerebellum, the basal ganglia, the thalamus, the hippocampus, the inferior parietal cortex, the frontal lobe, and, more specifically, the prefrontal cortex (PFC) are involved in upright standing maintenance (Bostan and Strick, 2011). Reduced activity in PFC is associated with better balance performance in healthy young adults compared to older adults, possibly due to higher efficiency in information processing (Lehmann et al., 2022). In addition, thanks to the numerous reciprocal projections from different brain areas, PFC is a strategic area for several functions such as motor conception, prediction, attention, decision-making, and memory (Sugihara et al., 2021). PFC also presents associations between sounds and motor responses (Huang and Brosch, 2020): in fact, the neurons of this cortical area dynamically establish a functional connection during auditory activities, showing that PFC is involved in the selection of the motor responses not only for visual stimuli but also for auditory stimuli. Listening to music is well-known to activate PFC areas that are involved in supporting executive functions, a set of higher-order neurocognitive processes that allow higher organisms to make choices and to engage in purposeful, goal-directed behavior (Suchy, 2009; Moriguchi and Hiraki, 2013), with a possible direct negative or positive influence on cognitive processes (Mansouri et al., 2017). However, to the author’s knowledge, no information about the neural mechanisms underlying PFC activity under different pure tone frequency conditions is available. Further investigation of this aspect could be crucial, especially in the clinical context where the early identification of cognitive deficits could be crucial for the realization of appropriate and timely interventions. In addition, clinicians should consider auditory cues and hearing loss in balance and fall-risk assessments to design tailored rehabilitation strategies, In the last 20 years, functional Near-InfraRed Spectroscopy (fNIRS) opened new fields of real-world neuroscience (Vidal-Rosas et al., 2023): fNIRS has been extensively applied in cognitive neuroscience in disease research (Ye et al., 2023) and in the field of gait and posture (Menant et al., 2020), considering its low susceptibility to motion artifacts (Stuart et al., 2018; Quaresima and Ferrari, 2019; Bishnoi et al., 2021; Yücel et al., 2021) and reliability (Stuart et al., 2019; Ayaz et al., 2022). fNIRS is a non-invasive vascular-based technique that enables real-time detection of concentration changes in oxygenated (O_2_Hb) and deoxygenated (HHb) hemoglobin of the cerebral microcirculation blood vessels. The coupling between neuronal activity and cerebral blood flow is at the basis of brain function, and fNIRS relies on this coupling to infer changes in neural activity, which is reflected by the blood oxygenation changes of the activated cortical region (i.e., the increase in O_2_Hb and the concomitant decrease in HHb). Despite fNIRS is effective in detecting cortical activations in response to different auditory intensities of a broadband noise stimulation (Weder et al., 2020), this technique could be appropriate in outlining the relationship between underlying cortical activity and pure tone auditory stimuli, the latter possibly linked to ecological warning/alarm signals, possibly processed by the frontal attentional network (Andrees et al., 2006). Moreover, postural responses to different sound frequencies need to be further explored due to the controversial existing results. Given these premises, the first aim of the study was to investigate PFC and postural sway responses to different auditory frequency stimuli in healthy young adults. The second aim was to correlate cerebral and postural activity to subjective pleasant and discomfort ratings to observe the eventual physiological influence on collected data. Lateral and medial regions of PFC activity, in fact, have been found associated with neural substrates that are primary emotion processing areas (e.g., amygdala), showing increased activation of right dorso-lateral PFC during down-regulation of negative emotions (Campbell-Sills et al., 2011). We therefore hypothesized that higher frequencies, given their use as warning cues, for instance in go/no go tasks (Straughn et al., 2009), would have been unpleasant for participants and, therefore, correlated with an increased postural sway and higher PFC activity compared to lower frequencies, especially in more challenging balance conditions.

## 2. Materials and methods

The study was approved by the Institutional Review Board of University of Rome “Foro Italico” (approval number: CAR 95/2021). All participants provided their written informed consent for participating in the study.

### 2.1. Participants

Twenty-two normal-hearing young adults, with a high-level of education, were enrolled in the study. To exclude left-handed subjects, and therefore possible consequent brain asymmetries (Sha et al., 2021), all participants completed the Edinburgh Handedness Inventory assessing hand dominance. This sample size complied with the minimum number of participants recommended by a power analysis purposely performed for a repeated measures design (16 subjects, effect size *d* = 0.7, *α* = 0.05) (Cohen, 1992). To be eligible, subjects were required to: (i) be between 18 and 40 years old; (ii) obtain a score in the hearWHO app over the minimum threshold of 50^1^; (iii) not have suffered from otitis or ear infections and/or severe vertigo episodes in the last 3 months; (iv) not exhibit any neurological, balance and/or vestibular disorders; (v) not present visual impairments (not correctible with lenses); (vi) not report motor and/or foot impairments (i.e., flat foot, plantar Fasciitis) which could influence the balance performance; (vii) not practice any contact sports (i.e., boxing) which can lead to chronic progressive brain damage; (viii) not assume caffeine the 2 h before the test and alcohol/drugs in the 24 h before the test.

### 2.2. Protocol

**Figure 1:**
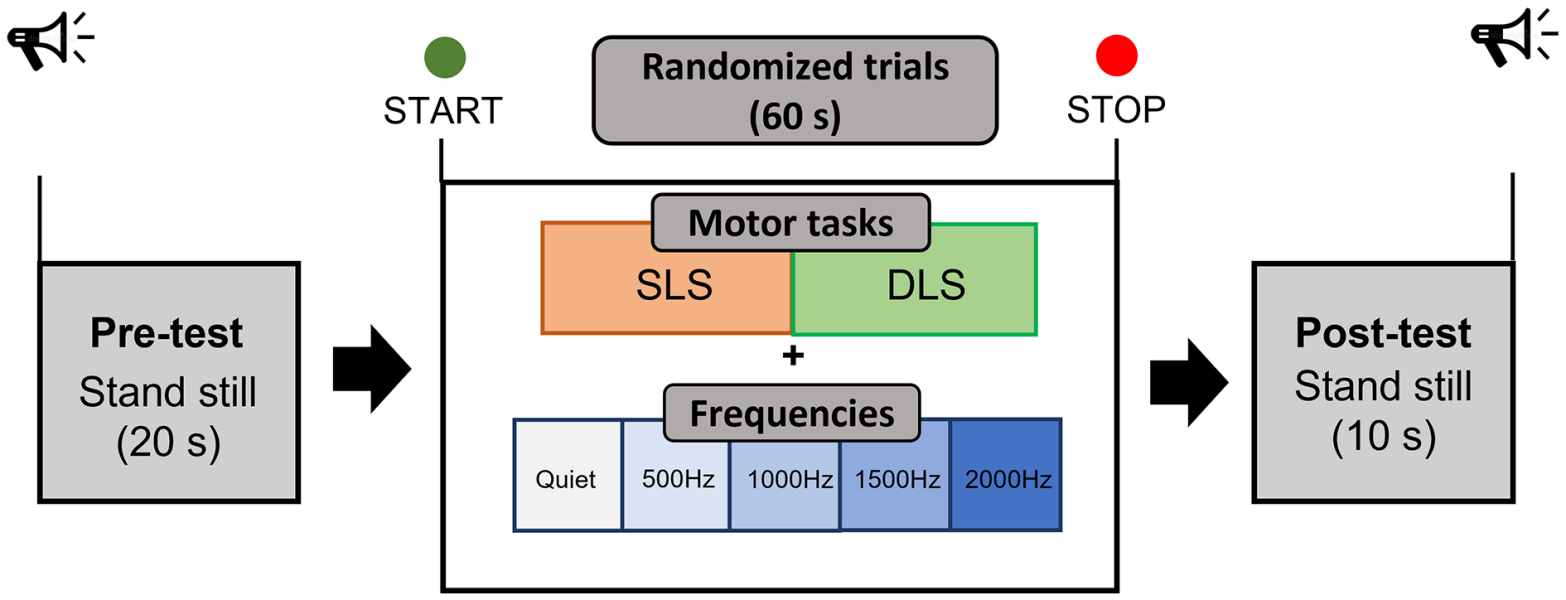
Experimental protocol. SLS = single-leg stance; DLS = double-leg stance.

Testing sessions have been performed in a quiet and dimly lit room of the Bioengineering and Neuromechanics Laboratory of the University of Rome “Foro Italico.” Prior to the study, participants were informed about the procedures and familiarized with the protocol. The familiarization phase was carried out 1 day before the study. Recruited participants performed a static single-leg stance and a double-leg stance test on a flat surface while looking at a fixed target on a white wall at participant’s eyes level, approximately located at 2-m distance, and while wearing sport comfortable shoes. Four auditory conditions, usually employed during pure tone audiometry testing (500, 1000, 1500, and 2000 Hz) were binaurally delivered through noise-isolating wireless headphones (Bose® SoundSport Free, 65 dB SPL, Framingham, MA, USA) and in quiet condition, for a total of 10 trials of 90 s each performed in a randomized order (20 s of initial static where participants were asked to assume the required position, 60 s of task execution used for the processing of the data, and 10 s of static in bipodalic condition at the end of the trial), as in Figure 1. During the baseline static period, participants were asked to relax to get fNIRS signals as stable as possible. A sound intensity of 65 dB was identified as suitable due to previous studies showing a decrease and an increase in O_2_Hb at lower (40 dB) and higher intensities (90 dB), respectively (Weder et al., 2020). Nonetheless, 65 dB SPL represents a common intensity level employed in auditory tasks (Cartocci et al., 2019). No information about the different sound frequencies has been provided to participants, but they were only instructed to “passively listen to a sound” while performing the two different balance tasks. Single-leg stance consisted of standing unassisted on the dominant leg, with the foot off the floor and maintaining a 90° angle between the knee and the thigh, without letting legs touch each other, and with the arms resting at the sides or on the hips. The double-leg stance consisted of standing feet shoulder-width apart in a comfortable position, with the arms resting at the sides. The feet position for both tests was marked on the floor to ensure intra-subject reliability among trials (McIlroy and Maki, 1997). A gentle push was adopted to determine the participant’s leg used to break the fall: however, it should be noted that recent evidence suggests that the leg’s dominance does not influence balance performance in unilateral stance (Schorderet et al., 2021). Both motor tasks have been intentionally carried out with open eyes only: in fact, the loss of the visual input could have forced the central nervous system to rely more on other inputs, with the visual loss possibly being compensated for in the brainstem (Zhong and Yost, 2013). After each trial with auditory stimuli, the perceived pleasantness (in the meaning of happiness/enjoyment) and discomfort (the state of being/not being relaxed) during the exposure to the stimuli were, respectively, assessed through two visual analogue scales (VAS) (Lichtenberg, 2010) of 10 cm each: one VAS concerned the pleasantness induced by the sound (0 = extremely unpleasant/100 = extremely pleasant), while the other one the discomfort induced by the sound (0 = no discomfort/100 = extreme discomfort). Participants were required to mark on the 10 cm scale the point that they felt representing their perception. The VAS score was then determined by measuring (in centimeters) the distance from the left end of the line to the point that participants marked.

### 2.3. Instrumentation and data processing

#### 2.3.1. fNIRS data acquisition and processing

**Figure 2:**
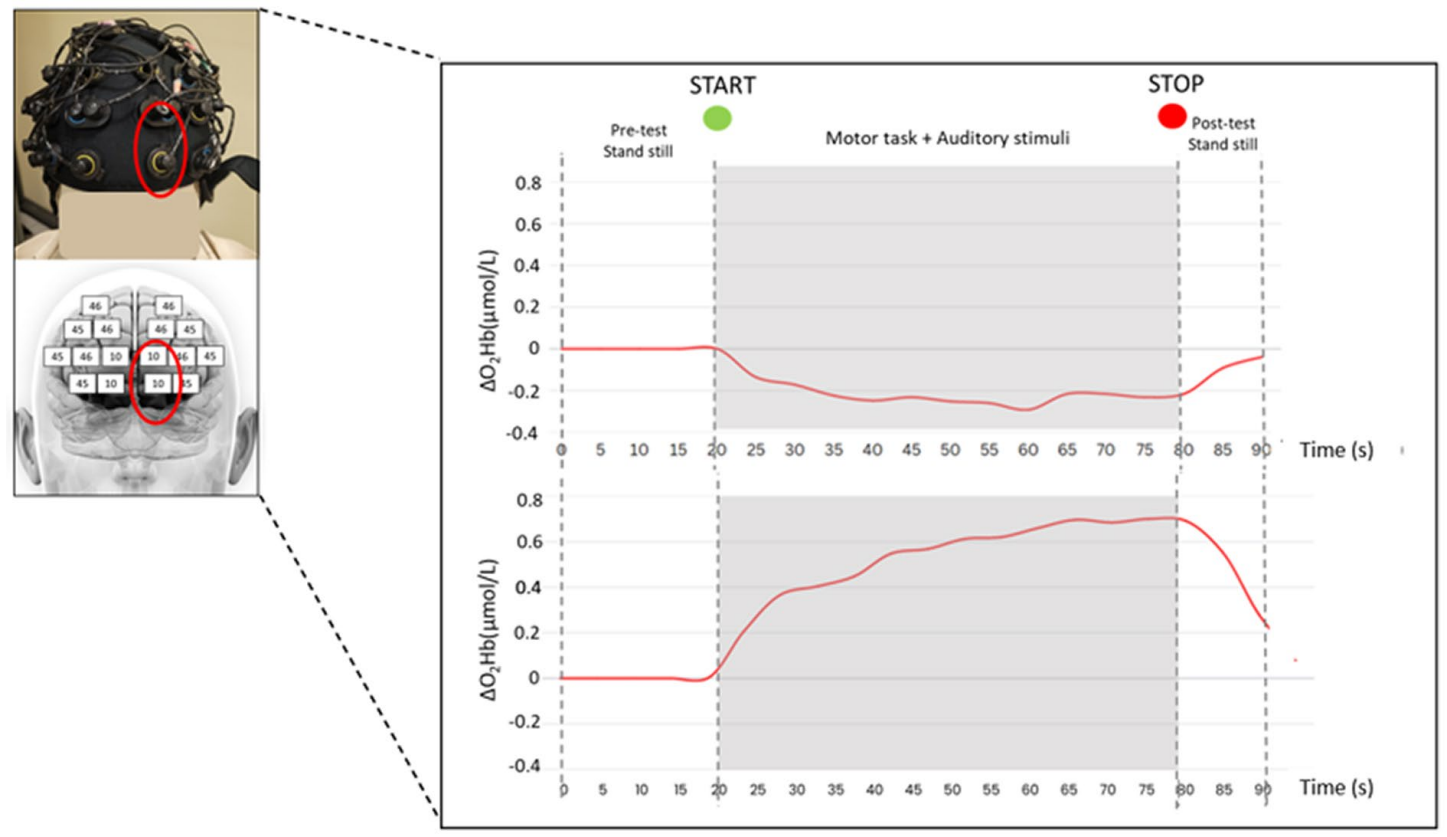
fNIRS head cap and a typical hemodynamic response (i.e., O_2_Hb concentration changes) measured over the PFC (one measurement point corresponding to BA10) of a representative subject in quiet condition during the 90 s trial in double-leg stance **(top panel)** and single-leg stance **(bottom panel)** tasks.

A 24-channels wearable continuous wave fNIRS system (Brite24, Artinis Medical Systems B.V., Elst, Netherlands) with dual-wavelength LED sources (760 and 850 nm) and 18 optodes (10 transmitters and 8 receivers, as in Figure 2) has been used. The system measures the changes optical densities that are converted into concentration changes in O_2_Hb (ΔO_2_Hb) and HHb (ΔHHb) by applying the modified Beer–Lambert low (Yücel et al., 2021). More specifically, enhanced brain activation induces an intensified blood flow in the investigated brain region, leading to an increase in O_2_Hb and a decrease in HHb concentration levels (Ferrari and Quaresima, 2012). Participants were instrumented with a soft neoprene head cap (2.5 mm thickness, sizes S-L: S, 53–55 cm; M, 55–57 cm; L, 57–59 cm) placed over the frontal regions of the forehead. The head cap was appropriately placed over the head in order to include the underlying PFC. In particular, the two frontopolar detectors collecting light at the bottom of the head cap were centered (according to the International 10–20 system for the electroencephalography electrode placement) on the Fp1 and Fp2 locations for the left and right side, respectively (Figure 2). The Montreal Neurological Institute coordinates of the optodes and the relative 18 measurement points were calculated using a probe placement method based on a physical model of the ICBM152 head surface (Cutini et al., 2011). The resulting matching Brodmann areas (BAs) of the measurement points were BA9, BA10, BA45, and BA46 (Lancia et al., 2018). Moreover, these BAs correspond to the major subregions of the PFC: the dorsolateral PFC (BAs 9 and 46) and the ventrolateral PFC (BA45). Data, sampled at 10 Hz, were collected with the Oxysoft software (version 3.2.70), while processing was performed with custom-written MATLAB codes and Homer2 software (MATLAB® 2019, The MathWorks, Inc., Natick, MA, USA). The first step was a visual inspection to identify and manually remove artifacts for each task; then, a low pass filter with a cut-off frequency of 0.1 Hz was applied to reduce physiological noise. To remove motion artifacts, a wavelet filter was used (Cooper et al., 2012; Brigadoi et al., 2014) as well as a principal component analysis filter with the recommended value of 80% of explained variance (Brigadoi et al., 2014). Finally, the filtered optical density signals were transformed into concentration changes of O_2_Hb and HHb. Sound- and task-induced related ΔO_2_Hb and ΔHHb were evaluated relative to the quiet standing O_2_Hb e HHb levels before each task (initial 20s) to control for transient effects of the signal. As also reported in previous studies, ΔO_2_Hb seems to be a more reliable parameter for measuring mobility-dependent changes in cortical oxygenation than ΔHHb (Miyai et al., 2001; Holtzer et al., 2011; Mirelman et al., 2014; Herold et al., 2017; Belluscio et al., 2021; Ayaz et al., 2022; Kinder et al., 2022): therefore, ΔO_2_Hb is here reported as primary outcome for PFC activation.

#### 2.3.2. IMUs data acquisition and processing

A wearable inertial measurement unit (IMU) (Opal, APDM, Portland, OR, USA, 128 Hz) was placed on participant’s pelvis (L5 level) and securely fixed with Velcro straps to avoid oscillations. The unit was aligned with the corresponding anatomical axes (antero-posterior: AP, medio-lateral: ML, and cranio-caudal: CC) following the procedure proposed by Bergamini et al. (2014). The resulting data was filtered with a Butterworth low-pass filter (4th order, 3.5 Hz). For our purposes, only AP and ML components were considered. All data processing was performed using Matlab®. The following parameters (Table 1) have been extracted from acceleration signals as in Mancini et al. (2012).

**Table 1:** Postural sway parameters.

RMS	Root mean square of acceleration data (m/s^2^)
Distance	Mean distance from center of trajectory (m/s^2^)
Sway path	Total length of acceleration trajectory (m/s^2^)
Mean velocity	√(∫𝐴𝑃)^2^ + (∫𝑀𝐿)^2^ (mm/s)
Mean frequency	Sway Path/(2∗𝜋∗𝑑𝑖𝑠𝑡𝑎𝑛𝑐𝑒∗𝑡𝑟𝑖𝑎𝑙 𝑑𝑢𝑟𝑎𝑡𝑖𝑜𝑛) (Hz)

AP, ML, Antero-posterior and medio-lateral accelerations.

Those indexes may provide deeper information on sway control: while higher mean frequency values correspond to more efficient postural control, the other parameters indicate a better performance when their values are low (Mancini et al., 2012).

### 2.4. Statistical analysis

Statistical analysis was performed using the IBM SPSS Statistics software (v23, IBM Corp., Armonk, NY, USA.; α level of significance = 0.05). The normal distribution of each parameter was verified using the Shapiro–Wilk test. Since most parameters were not normally distributed (*p* > 0.05), a Friedman Test was performed to assess differences among the different sound conditions in the PFC activity, postural sway parameters, and VAS results. Furthermore, after high and low frequencies have been averaged, a Mann–Whitney test for VAS results has been performed. For the *post hoc* analysis, the Wilcoxon Signed Rank test was used, while the Holm–Bonferroni correction was adopted to prevent type I error inflation due to multiple comparisons. In addition, Spearman R was computed for assessing correlations among O_2_Hb, biomechanical parameters, and VAS results.

## 3. Results

### 3.1. Participants

After the processing phase, the data of two participants have been excluded due to missing data: therefore, data from 20 subjects have been analyzed. Demographic and anthropometric participant’s characteristics have been reported in Table 2.

**Table 2:** Demographic and anthropometric participant’s characteristics.

Participant’s characteristics	
Sex [females-males]	11–9
Age [years]	24.4 (2.7)
Body Mass [kg]	66 (11.5)
Height [*m*]	1.71 (0.7)
Audiometric test [%]	72.8 (9.6)
Foot dominant side [left %]	65

Mean and standard deviations (in brackets) values are reported. Audiometric test result and foot dominant side is reported in percentage (%).

### 3.2. fNIRS and VAS results

**Figure 3:**
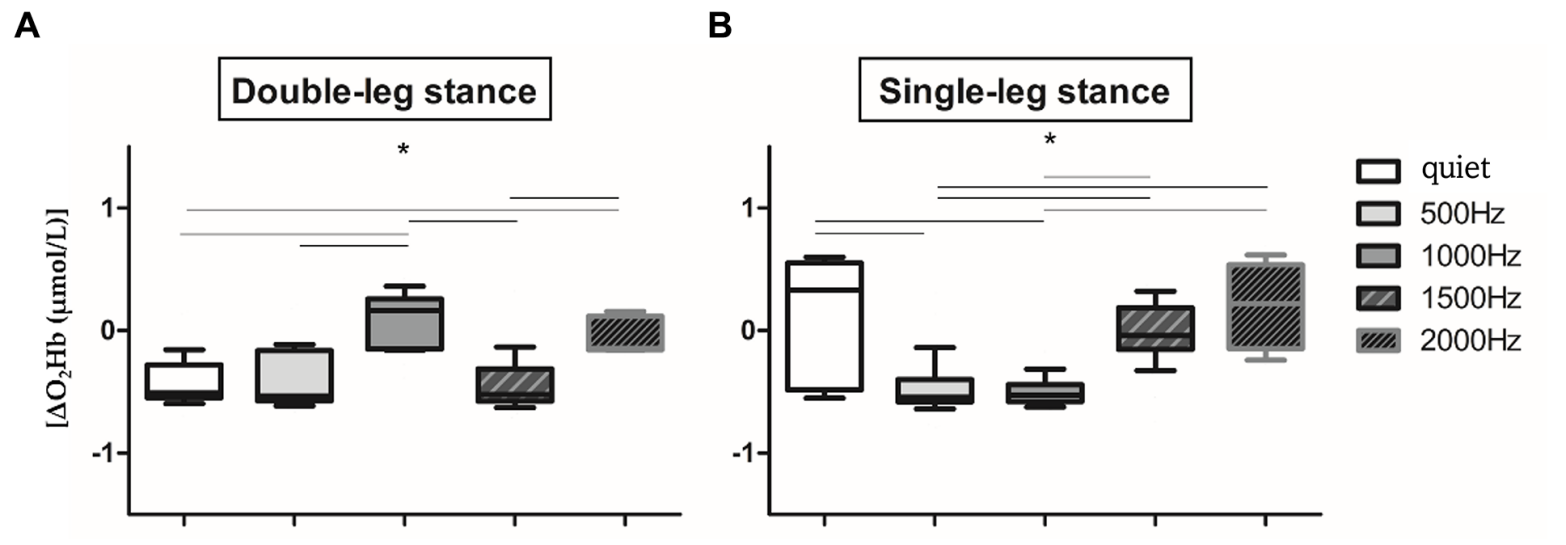
Whiskers plots report relative ΔO_2_Hb changes in double-leg stance (left panel, **A**) and single-leg stance (right panel, **B**) tasks. *Indicates statistically significant differences among auditory conditions (*p* < 0.005) after Holm-Bonferroni corrections.

Since no significant difference in O_2_Hb has been observed between the two hemispheres in all the proposed conditions (*p* > 0.05), right and left O_2_Hb values of the 60s signal have been averaged, as in Mirelman et al. (2014) and Belluscio et al. (2021). Therefore, from now on, results refer to the O_2_Hb of the entire PFC without distinction between right and left cortex. Several statistically significant differences have been found in the two proposed balance tasks in the different auditory conditions (Figures 3A,B). In detail, in the double-leg stance (Figure 3A) the maximum PFC activation has been found in the correspondence of 1000 Hz, with a slight activation at 2000 Hz and almost no activation (or de-activation) has been observed in the quiet and at the other frequencies. Significant differences have been found between the quiet condition and 1000 Hz and 2000 Hz (*p* < 0.001, 4,197 < Z < 5.251), 500 Hz and 1000 Hz (*p* < 0.001, Z = 5.184), 1000 Hz and 1500 Hz (*p* < 0.001, Z = 5.184), and 1500 Hz and 2000 Hz (*p* < 0.001, Z = 5.286). It is also possible to visually observe a reduced variability during the double-leg stance compared to the single-leg stance. In the single-leg stance task (Figure 3B) there is a PFC activation in the quiet condition, which is statistically significant with the 500 Hz (*p* < 0.001, *Z* = 4.287) and 1000 Hz conditions (*p* < 0.001, *Z* = 4.516). Other statistically significant differences have been found between 500 and 1500 Hz (*p* < 0.001, Z = 4.099), 500 and 2000 Hz (*p* < 0.001, *Z* = 5.137), 1000 and 1500 Hz (*p* < 0.001, *Z* = 4.509), and 1000 and 2000 Hz (*p* < 0.001, Z = 5.232).

**Figure 4:**
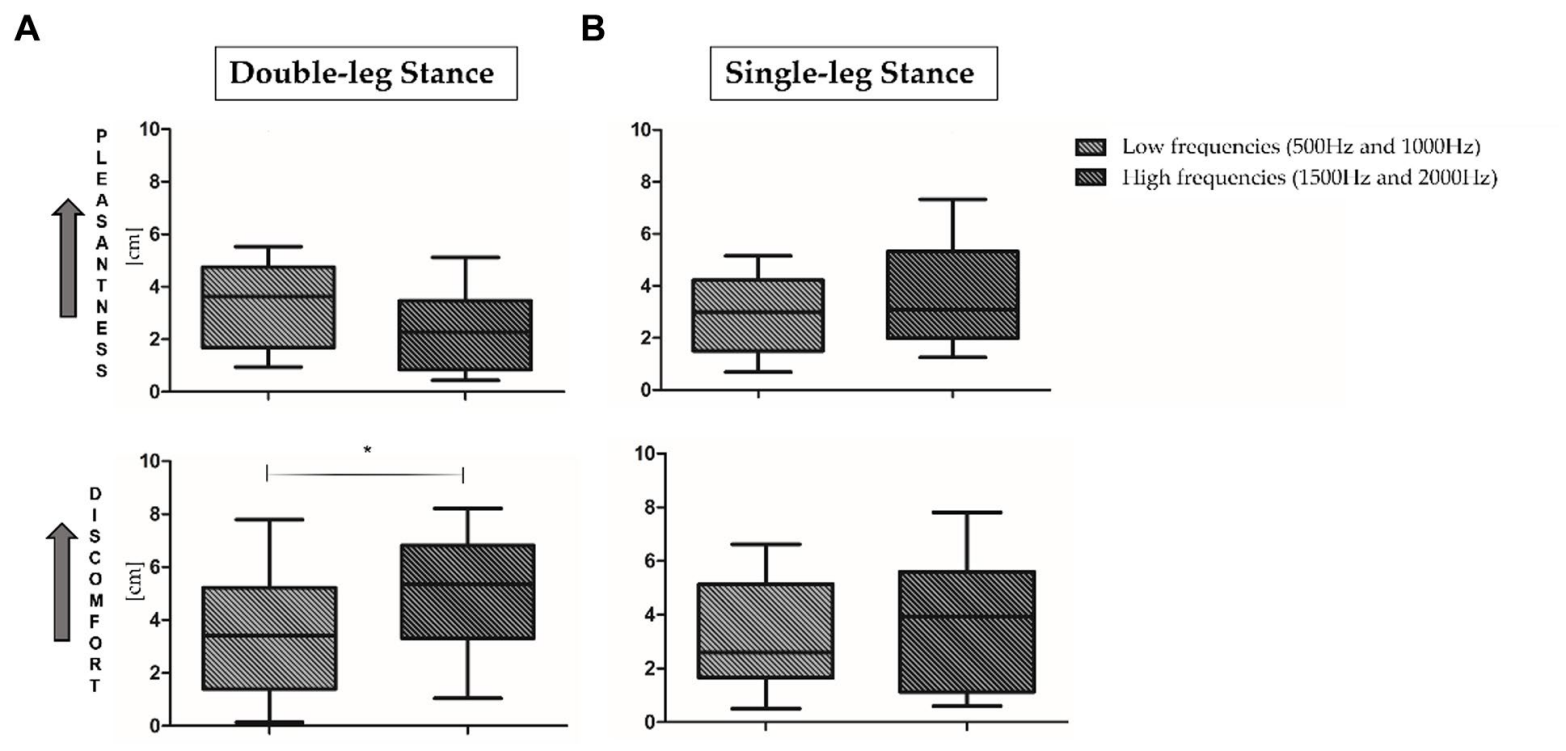
Visual analog scale (VAS) results in double-leg stance (left panel, **A**) and single-leg stance (right panel, **B**) tasks. 0 = minimum pleasantness/discomfort; 10 = maximum pleasantness/discomfort. *Indicates statistically significant differences between auditory stimuli. Low (500 and 1000 Hz) and high (1500 and 2000 Hz) frequencies have been averaged.

For what concerns VAS results, no statistically significant differences have been observed when comparing the four proposed frequencies. As proposed in the literature (Kiefer et al., 2004; Simpson, 2009), low frequencies (500 and 1000 Hz, top panel, Figure 4A) and high frequencies (1500 and 2000 Hz, bottom panel, Figure 4B) results have been averaged. As reported in Figure 4, during both motor tasks low frequencies seem to be more pleasant and less uncomfortable than high frequencies. In addition, a statistically significant increase of rated discomfort for high frequencies in comparison to low frequencies has been found in the double-leg stance (*p* = 0.03, Z = 2.092).

### 3.3. IMU results

**Figure 5:**
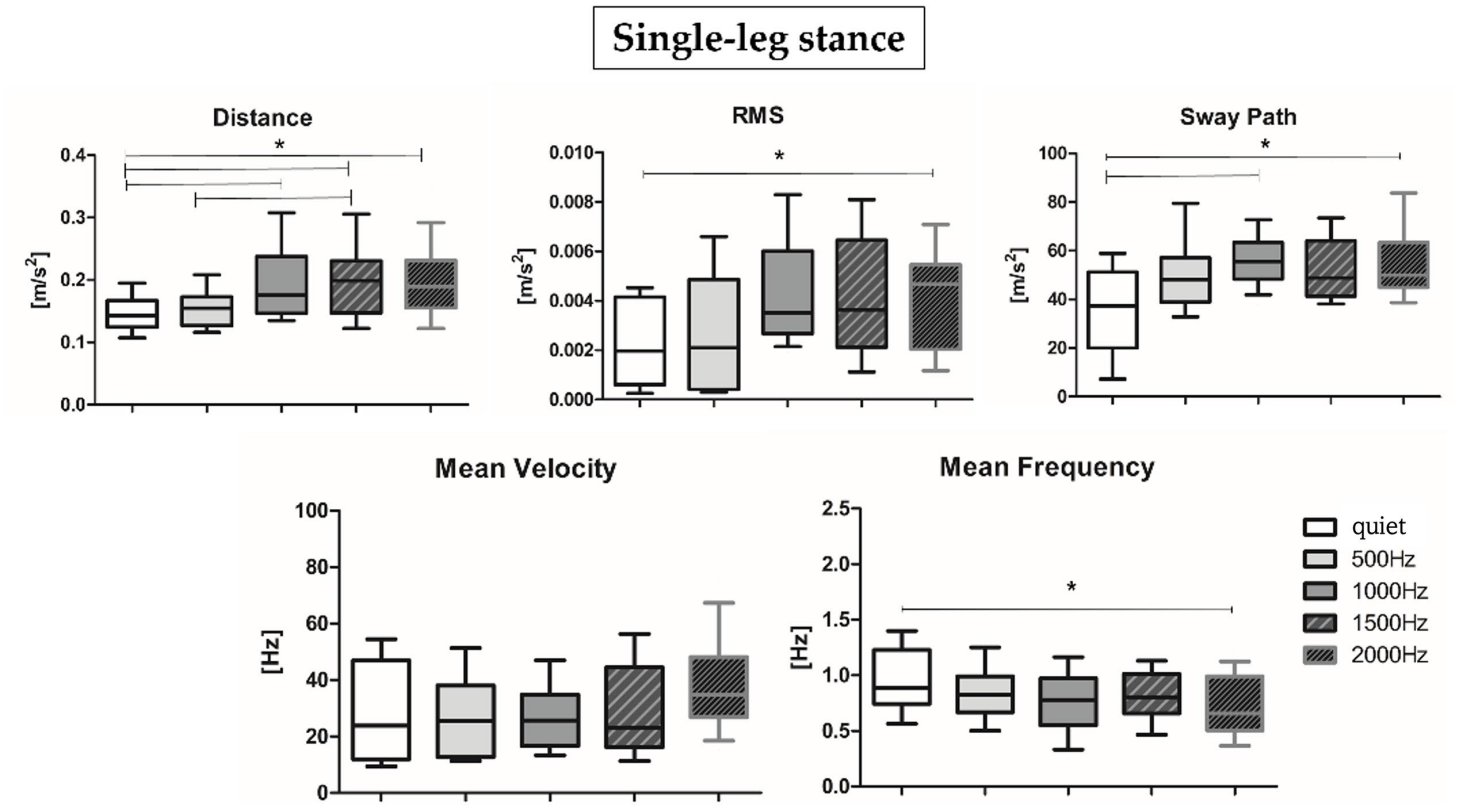
Median and interquartile values of postural parameters obtained from IMU for double-leg stance. *Indicates a statically significant difference (*p* < 0.05).

**Figure 6:**
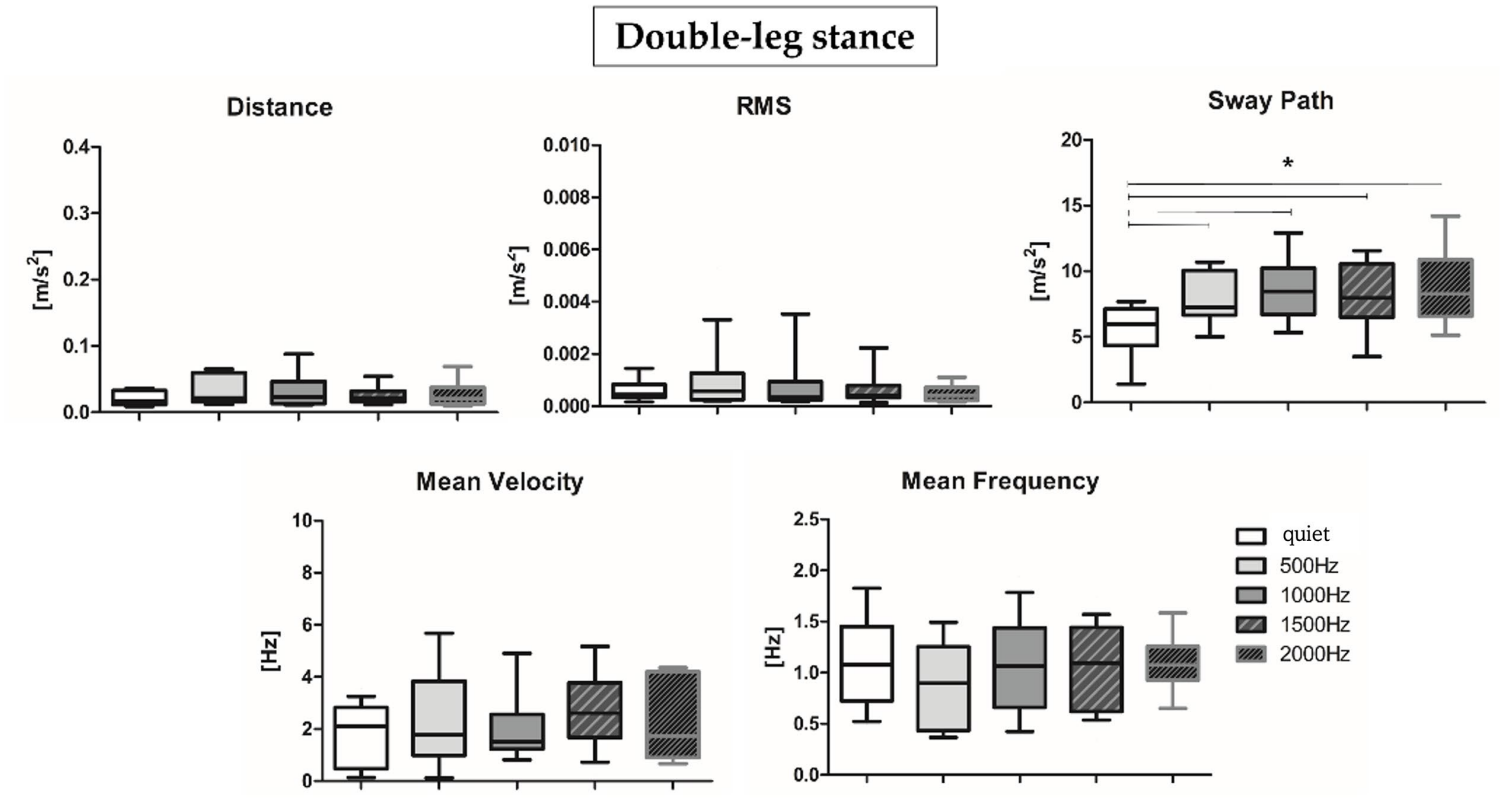
Median and interquartile values of postural parameters obtained from IMU for single-leg stance. *Indicates a statically significant difference (*p* < 0.05) after Holm-Bonferroni correction.

Results about postural parameters obtained from the IMU located at L5 level for double-leg stance (top panel, Figures 5A, 6A) and single-leg stance (bottom panel, Figures 5B, 6B) are displayed in Figures 5, 6, respectively. During the double-leg stance the sway path parameter presented statistically significant differences when comparing the quiet condition with all proposed auditory frequencies (*p* < 0.001, 0.4,097 < Z < 5.356). Auditory stimuli, whatever the frequency, increased the sway path, thus worsening balance performance. Several statistically significant differences have been observed during single-leg stance: the distance parameter showed statistically significant differences when comparing the quiet condition with 1000, 1500, and 2000 Hz, as well as when comparing 500 Hz and 1500 Hz (*p* < 0.001, 4,391 < Z < 5.251). For the RMS, a statistically significant difference has been observed between the quiet condition and 2000 Hz (*p* = 0.003, Z = 4.028), for the sway path between quiet and 1000 Hz (*p* = 0.002, Z = 4.856) and quiet and 2000 Hz (*p* = 0.001, Z = 4.287), while for the mean frequency between quiet and 2000 Hz (*p* = 0.001, Z = 4.128).

### 3.4. Correlation results

#### 3.4.1. IMU and O_2_Hb

Correlation results among IMU parameters and O_2_Hb at quiet condition and at the different auditory frequencies (500, 1000, 1500, and 2000 Hz) are reported in Tables 3, 4. During the double-leg stance (Table 3), positive correlations at 1000 Hz have been found between O_2_Hb and distance, and O_2_Hb and mean velocity, revealing that when PFC activation increases, also the distance and the mean velocity increase. During single-leg stance (Table 4), a negative correlation has been found at baseline condition between O_2_Hb and the sway path, suggesting that when the PFC activation increases, there is also a reduction in the sway path. In addition, a positive correlation has been observed at 2000 Hz between O_2_Hb and the mean velocity, meaning that increased PFC activation is accompanied by increased velocity.

**Table 3:** Double-leg stance correlation results among O_2_Hb and IMU parameters are displayed.

	Quiet	500 Hz	1000 Hz	1500 Hz	2000 Hz
	H_2_Ob				
RMS	−0.031	0.197	0.336	0.024	0.022
Distance	−0.254	0.093	0.716*	0.246	−0.166
Sway Path	−0.037	0.181	0.277	0.244	−0.257
Mean Frequency	0.092	−0.273	−0.405	0.271	0.181
Mean Velocity	−0.442	−0.360	0.611*	0.178	0.008

Positive correlations are displayed in blue and negative correlations in red color. O_2_Hb and postural parameters are correlated only for the same auditory condition. Color intensity is proportional to the strength of the correlation: red color identifies negative correlations, blue color positive correlations. *p < 0.05; **p < 0.01. O_2_Hb, oxygenated hemoglobin.

**Table 4:** Single-leg stance correlation results among O_2_Hb and IMU parameters are displayed.

	Quiet	500 Hz	1000 Hz	1500 Hz	2000 Hz
	H_2_Ob				
RMS	0.331	−0.095	−0.117	0.226	−0.006
Distance	0.189	0.155	0.114	0.033	-0.328
Sway path	-0.640*	0.251	-0.378	0.036	0.051
Mean frequency	-0.053	0.051	-0.584	0.128	0.341
Mean velocity	0.106	-0.218	-0.220	0.103	0.984**

Positive correlations are displayed in blue and negative correlations in red color. O_2_Hb and postural parameters are correlated only for the same auditory condition. Color intensity is proportional to the strength of the correlation: red color identifies negative correlations, blue color positive correlations. *p < 0.05; **p < 0.01. O_2_Hb, oxygenated hemoglobin.

#### 3.4.2. VAS

For the double-leg stance, statistically significant correlations have been found between the perceived pleasantness and discomfort and IMU parameters. In particular, for the 500 Hz tone stimulation a positive and a negative correlation between discomfort (*p* < 0.05, *R* = 0.594) and pleasantness (*p* < 0.05, *R* = −0.553) have been observed with respect to the sway path.

## 4. Discussion

The present study aimed at (i) investigating PFC activation and postural sway responses to different auditory frequency stimuli in healthy young adults and at (ii) assessing the correlation between these responses to pleasantness and discomfort rating. We would have expected to observe, at higher frequencies, an increase in terms of postural sway, accompanied by higher PFC activation, especially during the single-leg stance task, considered more challenging (Straughn et al., 2009; St George et al., 2021). Results partially supported our hypotheses: during the single-leg stance, auditory stimuli, especially those presented at higher frequencies, had a negative impact on the motor performance, but we did not observe an increment in terms of PFC activation or differences of subjective rated pleasantness and discomfort. More specifically, to safely maintain balance during the single-leg stance condition, the postural control system needs to reorganize the total body center of mass over a narrow base of support (Riemann and Schmitz, 2012): consequently, as expected, a slight activation of the PFC was shown in the quiet condition (Figure 3B, right panel). Accumulated evidence suggests that attentional ability plays a crucial role in postural control (Fujita et al., 2016): therefore, PFC activation may reflect attentional processing during the single-leg stance task and preparation for ankle joint movement to prevent body sway. In addition, while performing the single-leg stance task, a slight PFC activation has been observed also at 2000 Hz, a frequency often employed for auditory tasks related to attention (i.e., oddball tasks) and in conditions characterized by attentional deficits (i.e., Attention Deficit Hyperactivity Disorder, ADHD) (Dolu et al., 2019). In particular, in the cited reference, it has been found that healthy children presented higher (right) PFC activation in comparison to ADHD mates, supporting the hypothesis of an involvement of the PFC in the attentional response to such auditory stimuli (Altinkaynak et al., 2018). Moreover, during dual tasks employing 2000 Hz stimuli, it has been found an effect of such tone on the cerebral activity, as indexed by the variation of alpha and beta electroencephalographic rhythms (Beurskens et al., 2016). Therefore, the sum of this evidence supports the hypothesis of the physiological role of PFC activation in response to 2000 Hz during the execution of attentional and motor tasks. During the double-leg stance condition, considered less challenging than the single-leg stance, we observed a PFC de-activation in the quiet condition. In the double-leg stance condition subjects displayed a PFC activation at 1000 Hz and a slight activation at 2000 Hz, while at 500 and 1500 Hz we observed a de-activation (Figure 3A, left panel). As previously reported, this result could be related to the higher response in terms of cerebral activation found for the higher perceived loudness as stated in the equal loudness perception curves (Weder et al., 2020). According to such curves, below 1000 Hz the ear is less sensitive to low frequencies, resulting in a decreased perceived loudness; at 1000 Hz, there is a point of equality between sound intensity and perceived loudness; above 1000 Hz, it is possible to observe a slight decrease in the perceived loudness, but from 2000 to 5000 Hz there is a range of increased loudness, corresponding to the maximum sensitivity region for the human ear, associated with the resonance of the auditory canal. The sum of this evidence could explain the increase in terms of PFC activity at 1000 and 2000 Hz, but lower activation at 500 and 1500 Hz, because, respectively, corresponding to points of higher and lower perceived loudness of tones. In terms of motor performance, the presence of auditory stimulation produced a negative effect on the overall performance during the single-leg stance task (Figure 4B). More in detail, the worsening in the postural performance is particularly evident when subjects are exposed to higher frequencies (1000, 1500, and 2000 Hz) when compared to the quiet condition. These results confirm how, due to the shape of the external auditory canal, the auditory sensitivity of normal hearing humans is greater at frequency signals of 1000–5000 Hz (Ziayi Ghahnavieh et al., 2018) and may suggest that this frequency band would interfere with the vestibular system, reducing its capacity to maintain balance. However, it should be noted that our subjects were not asked to close their eyes during the tests, so they were able to use their visual inputs, other than the other systems, as an alternative source for balance control. Furthermore, the psychomotor reaction elicited at 2000 Hz, in addition to being due to an innate sensitivity from the inner ear, could be due to an alarm reaction triggered by the auditory stimulus. In fact, frequencies between 1000 and 2000 Hz are often used in literature to refer to danger/alarm situations (Laroche and Lefebvre, 2014). Furthermore, official guidelines^2^ indicate that the dominant frequency of a fuel reserve alarm in automobiles should be between 700 and 2800 Hz (Vaillancourt et al., 2013). On the other hand, while performing the double-leg stance task, the administration of auditory frequencies seems to not influence the motor performance, with the only exception of the sway path (Figure 6): this parameter, in fact, showed statistically significant differences between the quiet condition and all the administered auditory frequencies, indicating that it is not a specific frequency which affects sway path, but more in general the administration of a sound. For what concerns VAS results, the rating of perceived pleasantness decreased, and discomfort increased, as the frequency of sound increased, even if reaching a statistically significant difference between low frequencies (average of 500 and 1000 Hz) and high frequencies (average of 1500 and 2000 Hz) only in the double-leg stance for the discomfort (Figure 4). This could be explained by the maximum loudness perception of frequencies of 2000 Hz and above, as stated by the equal loudness curves. This could be more evident in the double-leg stance condition because of a lower general cognitive engagement in this task, less demanding than the single-leg stance, therefore leaving more cognitive resources for the perception and evaluation of the sounds, as suggested by Kahneman’s Capacity Model of Attention (Kahneman, 1973), already applied to listening by Pichora-Fuller et al. (2016). Interesting results have been obtained also when considering correlation analyses: O_2_Hb showed almost no clear correlations with postural sway parameters at the different frequencies, with the only exception of a positive correlation between O_2_Hb and the mean velocity at 2000 Hz (Table 3). This could be due to a neurophysiological brain response to the most discomfortable information that needs to be processed. Furthermore, only for the easier condition, the double-leg stance, some correlations have been evidenced among postural parameters and subjective ratings of perceived pleasantness and discomfort. Concerning the lowest frequency included in the study, 500 Hz, together with an increase in the sway path parameter we observed an increase in the perceived discomfort and a decrease in the perceived pleasantness. Noteworthy, 500 Hz frequency has been found to elicit vestibular evoked potentials in healthy adults (Govender et al., 2020). The increase in the perceived discomfort and, on the other hand, the decrease in the perceived pleasantness, could therefore be connected to a certain degree of perception of swaying, as supported by the higher discomfort rated by patients with vestibular diseases in comparison to healthy controls (Andersson et al., 1998). For what concerns the relationship between PFC activation and VAS, our results seem to support the valence asymmetry hypothesis: since we did not observe differences between right and left hemispheres, a bilateral frontotemporal activation was observed in response to negatively valenced auditory stimuli, confirming that the left hemisphere is specialized for the processing of positive emotions and the right hemisphere is specialized for the processing of negative emotions (Takeda et al., 2016). Further investigations should be performed in order to confirm this hypothesis. In conclusion, results obtained in the present study prove that specific sound frequencies play a significant role in cognitive resources recruitment and in the regulation of postural control. Future studies should involve people with pathological medical conditions, such as those who suffered of vestibular dysfunctions related to cochlear implantation (Parietti-Winkler et al., 2015): the insertion may in fact alter the inner ear and induce vestibular disorders like vertigo, dizziness, or imbalance and they could benefit from the postural evaluations in the follow-up of the cochlear implantation. It would also be interesting to expand the study to elderly people, in which structural and functional changes in the auditory system due to aging can limit speech comprehension (Gates and Miller, 2005) and would therefore benefit of specific auditory training. Further studies should also consider the possibility of using the proposed integrated approach to identify endogenous frequencies that may be targeted to improve postural control by delivering brief auditory stimuli at specific frequencies.

## 5. Study limitations

Different patterns of PFC activation have been displayed based on the task complexity: however, an aspect that should be taken into consideration is that all involved subjects were only instructed to “passively attend” the presented stimuli, not giving them information about the possibility of receiving sounds of different frequencies. It is known that prompting attention to a specific sound increases the activation induced by that sound (Weder et al., 2020): the difference in activation between low and high frequencies could have been therefore higher if some subjects paid more attention to the stimuli with the higher loudness or, vice versa, lower if they paid stronger attention to the stimulus with lower loudness. However, we believe that instructing subjects about the different frequencies could have directed their attention towards specific stimuli and eventually increase their arousal, possibly interfering with the aim of investigating the ecological modulation of environmental sounds to balance tasks. Moreover, we employed only four auditory frequencies, chosen on the base of usual pure tone audiometry, however it could be interesting to investigate further frequencies, especially higher ones. It should also be considered that Another aspect which should be considered is that participants were not assessed with official audiometric tests, but only through a WHO App; nonetheless, we believe that involved subjects did not present hearing deficits since they were all young, healthy, and involved in agonistic sport activities, therefore commonly subjected to clinical screening. It should also be noted that it was not possible to measure extracerebral tissue influences on fNIRS measurements, like the effect of the changes of scalp blood flow, since the device was not equipped with short-separation channels. However, the heart rate was measured by pulse oximetry during the different trials, and it was found almost unchanged in each subject; in addition, participants were asked to not move the head/look at their feet to avoid possible changes in the scalp blood flow. Therefore, authors are confident to speculate that the scalp blood flow interference can be considered negligible among the trials and did not influence the fNIRS findings.

## 6. Conclusion

Although it is certainly true that the hearing system does not have the same importance in balance control as vestibular, somatosensory, or visual afferents, the role of the acoustic stimulation in the maintenance of postural stability should be further considered. Furthermore, it seems to exist a relationship between PFC activation and pleasant/comfortable sounds, confirming the role of the PFC in emotional recognition and processing. The combination of information that it’s possible to obtain from fNIRS signals and postural parameters could perhaps support a more sensitive and effective early detection of persons with a high risk for falls or to develop cognitive diseases. In the clinical practice this, in turn, may allow an early onset of therapeutic interventions and an effective monitoring of intervention programs, supporting clinicians in the decision-making process.

## Data availability statement

The raw data supporting the conclusions of this article will be made available by the authors, without undue reservation.

## Ethics statement

The studies involving human participants were reviewed and approved by Institutional Review Board of University of Rome “Foro Italico” (approval number: CAR 95/2021). The patients/participants provided their written informed consent to participate in this study.

## Author contributions

VB, GC, and GV: conceptualization. VB, MF, VQ, and GV: methodology. VB, PDF, TT, MF, and VQ: software. VB and GV: validation. VB, PDF, GC, and TT: formal analysis. VB, PF, and TT: investigation. MF, VQ, and GV: resources. VB, GC, and BI: data curation. VB: writing – original draft preparation. VB, GC, TT, PDF, BI, MF, VQ, and GV: writing – review and editing. GC, MF, VQ, and GV: supervision. VB, PDF, and TT: project administration. All authors have read and agreed to the published version of the manuscript.

## Conflict of interest

GC and BI were employed by the company BrainSigns Ltd.

The remaining authors declare that the research was conducted in the absence of any commercial or financial relationships that could be construed as a potential conflict of interest.

## Publisher’s note

All claims expressed in this article are solely those of the authors and do not necessarily represent those of their affiliated organizations, or those of the publisher, the editors and the reviewers. Any product that may be evaluated in this article, or claim that may be made by its manufacturer, is not guaranteed or endorsed by the publisher.

## Footnotes

^1^Available at: https://www.who.int/teams/noncommunicable-diseases/sensory-functions-disability-and-rehabilitation/hearwho#:~:text=The%20hearWHO%20app%20is%20based,their%20hearing%20status%20over%20time (Accessed March 25, 2023).

^2^Available at https://www.sae.org/standards/content/j994_198503/ (Accessed 16/02/2023).
